# Tetracycline-inactivating enzymes from environmental, human commensal, and pathogenic bacteria cause broad-spectrum tetracycline resistance

**DOI:** 10.1038/s42003-020-0966-5

**Published:** 2020-05-15

**Authors:** Andrew J. Gasparrini, Jana L. Markley, Hirdesh Kumar, Bin Wang, Luting Fang, Sidra Irum, Chanez T. Symister, Meghan Wallace, Carey-Ann D. Burnham, Saadia Andleeb, Niraj H. Tolia, Timothy A. Wencewicz, Gautam Dantas

**Affiliations:** 1 The Edison Family Center for Genome Sciences & Systems Biology, Washington University School of Medicine, St. Louis, MO 63110 USA; 2 Department of Chemistry, Washington University, St. Louis, MO 63130 USA; 3 Department of Molecular Microbiology, Washington University School of Medicine, St. Louis, MO 63110 USA; 4 Laboratory of Malaria Immunology and Vaccinology, National Institute of Allergy and Infectious Diseases, National Institutes of Health, Bethesda, MD USA; 5 Department of Pathology and Immunology, Washington University School of Medicine, St. Louis, MO 63110 USA; 6 Atta ur Rahman School of Applied Biosciences, National University of Sciences and Technology, Islamabad, Pakistan; 7 Department of Pediatrics, Washington University School of Medicine, St. Louis, MO 63110 USA; 8 Department of Medicine, Washington University School of Medicine, St. Louis, MO 63110 USA; 9 Department of Biomedical Engineering, Washington University, St. Louis, MO 63130 USA

**Keywords:** Antimicrobial resistance, X-ray crystallography, Enzymes, Metagenomics

## Abstract

Tetracycline resistance by antibiotic inactivation was first identified in commensal organisms but has since been reported in environmental and pathogenic microbes. Here, we identify and characterize an expanded pool of *tet(X)*-like genes in environmental and human commensal metagenomes via inactivation by antibiotic selection of metagenomic libraries. These genes formed two distinct clades according to habitat of origin, and resistance phenotypes were similarly correlated. Each gene isolated from the human gut encodes resistance to all tetracyclines tested, including eravacycline and omadacycline. We report a biochemical and structural characterization of one enzyme, Tet(X7). Further, we identify Tet(X7) in a clinical *Pseudomonas aeruginosa* isolate and demonstrate its contribution to tetracycline resistance. Lastly, we show anhydrotetracycline and semi-synthetic analogues inhibit Tet(X7) to prevent enzymatic tetracycline degradation and increase tetracycline efficacy against strains expressing *tet(X7)*. This work improves our understanding of resistance by tetracycline-inactivation and provides the foundation for an inhibition-based strategy for countering resistance.

## Abstract (web summary)

Gasparrini et al. show that Tet(X7) contributes to tetracycline resistance in a clinical *Pseudomonas aeruginosa* isolate and demonstrate the use of a Tet(X) inhibitor to circumvent resistance. This study highlights the importance of *tet(X*)-mediated antibiotic resistance and inhibition-based strategies for countering it.

## Introduction

Benign environmental microbes are ancient and diverse reservoirs of antibiotic-resistance genes (ARGs), and are the likely evolutionary progenitors of much clinical resistance^1–5^. Nonpathogenic bacteria from habitats as varied as soils and healthy human guts have exchanged multidrug-resistance cassettes with globally distributed human pathogens^2^. With widespread antibiotic use in clinical and agricultural settings, there is strong selective pressure for pathogens to acquire resistance genes with novel activities and substrate specificities from environmental resistomes^6^. The discovery and characterization of environmental resistance genes with novel activities before they are acquired by pathogens can help lessen their potential clinical impact and inspire proactive approaches to address emerging resistance early in antibiotic development and improve understanding of the ecology, evolution, and transmission of resistance genes across habitats^4,7^. Furthermore, understanding the evolutionary origins, genetic contexts, and molecular mechanisms of antibiotic resistance is critical to devising strategies to curb the spread of resistant organisms and their ARGs, and for sustainable development of new antimicrobial therapies.

While tetracycline resistance most frequently occurs via efflux or ribosomal protection^8^, enzymatic detoxification of tetracycline was first reported in 1988^9,10^. This mechanism of resistance, originally detected in the commensal *Bacteroides fragilis*, has since been identified in broader commensals, environmental microorganisms, and pathogens. In 2015, we substantially expanded the catalog of “tetracycline destructases” by identifying a family of tetracycline-inactivating enzymes, (Tet(47)–Tet(55)), from functional selections of environmental metagenomes^11^. Based on sequence homology to these soil-derived enzymes, we identified an additional enzyme, Tet(56), as a previously uncharacterized tetracycline resistance determinant in *Legionella longbeachae*. These enzymes are structurally and functionally homologous to Tet(X)^12^, the flavin-dependent monooxygenase (FMO) originally discovered in *Bacteroides fragilis*^13^. Recently, we described the structural details of two complementary inhibitory mechanisms for the soil-derived tetracycline-inactivating enzymes: competitive inhibition by blockade of substrate binding, and mechanistic inhibition by restraining FAD cofactor dynamics^12^. In 2019, two plasmid-encoded variants of Tet(X), named Tet(X3) and Tet(X4), were discovered in Enterobacteriaceae and *Acinetobacter* strains isolated from animal and human sources. The Tet(X3)- and Tet(X4)-containing plasmids were widely dispersed, transferable, and stable in human pathogens, and conferred high levels of resistance (up to 128-fold increase) against all tetracycline antibiotics, including latest-generation tigecycline, eravacycline, and omadacycline, in antibiotic susceptibility assays and murine infection models^14,15^. Given that tetracycline antibiotics have been used clinically for seven decades and are currently widely deployed in agricultural settings^16^, it is likely that anthropogenic emissions have driven spread of Tet(X)-like FMOs that now threaten the clinical efficacy of next-generation tetracycline antibiotics.

In human pathogens, tetracycline resistance was thought until recently to occur almost exclusively by ribosomal protection or antibiotic efflux^8^. Eravacycline and omadacycline were developed, in part, because the synthetic scaffold modifications of the D-ring overcome these traditional clinical resistance mechanisms, similar to tigecycline. It is noteworthy that tigecycline was approved by the FDA in 2005 and saw somewhat limited use until the appearance of infections caused by multidrug-resistant (MDR) Gram-negative bacteria, including carbapenem-resistant Enterobacteriaceae^17^ and recalcitrant ventilator-associated pneumonia^18^, complicated urinary tract infections, and complicated intra-abdominal infections^19^. Tet(X) and homologs conferring resistance to tigecycline, eravacycline, omadacycline, and all other classes of tetracycline antibiotics have now been discovered to be present in carbapenem and colistin-resistant MDR organisms harboring the *bla*_NDM-1_ and *mcr-1* genes, respectively, creating a scenario of pan-antibiotic resistance emerging in Gram-negative pathogens^14,15^. The widespread distribution of *tet*(X)-like genes capable of covalent inactivation of tetracycline scaffolds threatens the future clinical efficacy of this drug class in the same way consecutive generations of aminoglycosides, amphenicols, and beta-lactams have become vulnerable to enzymatic inactivation as a dominant resistance mechanism^20^.

All three types of tetracycline resistance have evolutionary origins in the environment, but are now found widely distributed in commensal and pathogenic bacteria^8^. Flavoenzymes, including Tet(X)-like FMOs, are an abundant and diverse enzyme family and display a proclivity for horizontal transfer and gene duplication, allowing them to spread between species and acquire novel functionality^21^. Thus, these genes are candidates for dissemination, potentially compromising new tetracycline antibiotics and motivating surveillance of the prevalence and abundance of this gene family across microbial habitats. The soil tetracycline-inactivating enzymes and Tet(X)-like FMOs have low overall sequence identity (20.1% mean ± 1.2% s.d. percent amino-acid identity between Tet(X) and soil-derived tetracycline-inactivating enzymes) making ARG prediction difficult and functional validation of putative ARGs necessary. Structural and biochemical characterization of the soil tetracycline-inactivating enzymes have revealed novel structural features including an extra C-terminal helix that plays a role in active site gating and substrate selectivity and mechanistically distinct oxidation patterns leading to resistance for soil tetracycline-inactivating enzymes (oxidations at C11a and C1) and Tet(X) (oxidation at C11a)^12^. The soil tetracycline-inactivating enzymes detoxify naturally occurring first-generation tetracyclines, such as chlortetracycline and oxytetracycline, but fail to oxidize D-ring substituted analogs, including tigecycline^12^. Tet(X)-like homologs possess a constitutively open active site that accommodates D-ring substituted substrates, such as tigecycline, eravacycline, and omadacycline^12,14,15^, making Tet(X) homologs a clinical threat for these last-generation tetracyclines. Single amino-acid mutations in Tet(X) have been shown to provide gain-of-function under tigecycline selection through more efficient oxidative inactivation^22^. Thus, monitoring broadly for Tet(X) homologs, even single amino-acid point mutants, and understanding the evolutionary connection with the soil tetracycline-inactivating enzymes is critical for proactively managing this emerging resistance mechanism through optimization of next-generation tetracycline structures and the development of effective inhibitor combinations that overcome resistance by inactivation^23^.

To this end, we sought to characterize the tetracycline resistome across environmental and human-associated metagenomes. We used functional metagenomic selections, wherein the heterologous expression bottleneck allows only cloned fragments with a functional resistance gene to be sequenced and assembled^2^. Annotation of all open-reading frames in the selected fragments, using similarity to known resistance protein families (including remote homologs via hidden Markov models), enable identification of the gene likely to be responsible for the resistance phenotype in recombinant *E. coli*^24^. We previously employed this sequence- and culture-independent approach to identify the tetracycline-inactivating enzymes from 18 grassland and agricultural soil metagenomes^11^. Here, we expand that approach to analyze 244 additional metagenomes to ask whether novel tetracycline-resistance elements are found in other habitats, perhaps with different or expanded substrate range and specificity. We find genes encoding tetracycline-inactivating enzymes are widespread in diverse microbial communities, but cluster by habitat of origin and resistance phenotypes also correlate with microbial habitat. We show that tetracycline-inactivating enzymes identified in the human gut metagenome confer resistance to all tetracycline antibiotics tested. Furthermore, we describe in detail Tet(X7), a tetracycline-resistance enzyme recovered from a human gut metagenome that confers resistance to tigecycline, omadacycline, and eravacycline. We characterize the phenotypic resistance profile, solve a crystal structure, and reveal the biochemical basis of Tet(X7) degradation of tetracyclines. We identify Tet(X7) in a clinical isolate of *Pseudomonas aeruginosa* from a cystic fibrosis patient, further supporting the clinical arrival of tetracycline resistance by inactivation. Lastly, we show that inhibition of Tet(X7) with anhydrotetracycline or analogues prevents tetracycline degradation and rescues antibiotic efficacy. Our results emphasize the need for surveillance of novel resistance determinants to antimicrobial agents in development to counter emerging antibiotic resistance.

## Results

### Identification of tetracycline inactivation across habitats

**Fig. 1:**
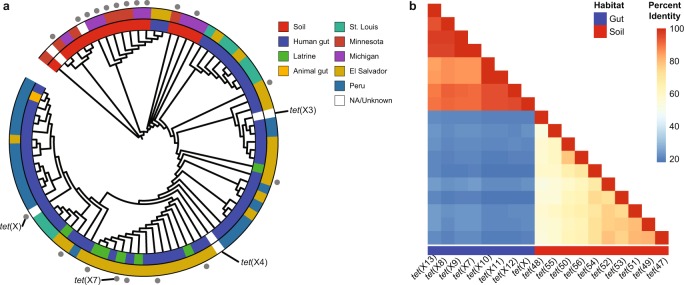
Diversity in tetracycline-inactivating enzyme sequence corresponds to microbial habitat of origin. **a** Sixty-nine tetracycline inactivators were identified from tetracycline and tigecycline selections of diverse metagenomic libraries. Genes selected for further analysis are indicated by gray dots at branch tips, and clinically implicated *tet*(X) variants are labeled. **b** Percent amino-acid identity heatmap indicates greater relative sequence diversity in the soil-derived set compared with the gut-derived set, but low identity between these two sets.

We sought to better understand the prevalence of tetracycline inactivation by FAD-dependent oxidoreductases as a resistance mechanism across habitats. Tetracycline resistance by inactivation is relatively uncommon as compared with efflux or ribosomal protection, so current resistance databases are biased toward these latter two mechanisms^24^. Functional metagenomics circumvents limitations imposed by identifying resistance via sequence similarity to a database^2^. We conducted a retrospective analysis of functional metagenomic libraries that had previously been selected on tetracycline and tigecycline^2,3,25–28^ [GenBank accession numbers: JX009202-JX009380, KJ691878-KJ696532, KU605810-KU608292, KF626669-KF630360, KX125104-KX128902, KU543693-KU549046]. These included libraries constructed from 53 soil samples, 176 human gut microbiota, 2 animal gut microbiota, and 13 latrine samples, and encompassed a total of 912 Gb of metagenomic DNA. By using a functional metagenomic approach, we explicitly limit our search space to microbial sequences that have an associated tetracycline or tigecycline-resistance phenotype. We specifically focused on putative tetracycline inactivators in these selections by searching for open-reading frames annotated as encoding an FAD-binding domain. In this manner, we identified 69 potential tetracycline-inactivating enzymes in addition to the 10 tetracycline-inactivating genes from soil metagenomes that had previously been described^11^ (Fig. 1a). We found that these genes formed two distinct clades that were correlated with habitat of origin. There was greater sequence diversity within the soil-derived subset (mean ± s.d. percent amino-acid identity of 66.5% ± 9.4%) compared with the gut-derived subset (mean ± s.d. percent amino-acid identity of 91.4% ± 4.7%), but low identity between the two habitats (mean ± s.d. percent amino-acid identity of 20.8% ± 1.5%, Fig. 1b).

**Fig. 2:**
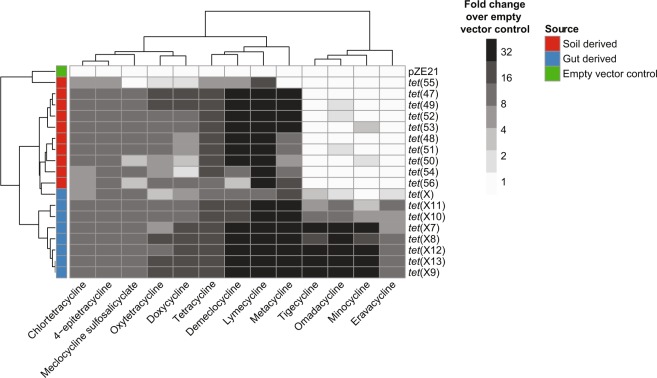
Phenotypic profiles of heterologously expressed tetracycline-inactivating enzymes correlate with habitat of origin. Antimicrobial susceptibility testing was performed by microbroth dilution on a subset of predicted tetracycline inactivators. We observed broad high-level resistance to tetracyclines within this class. Resistance phenotypes clustered by microbial habitat of origin, with resistance to latest-generation tetracyclines (e.g., minocycline, tigecycline, eravacycline, and omadacycline) discriminating between habitats. Each box represents the consensus MIC value determined for three independent trials. Dendrogram represents hierarchical clustering of resistance phenotypes.

We selected eight genes from this set for further analysis and subcloned them from their metagenomic source into a pZE21 expression vector and transformed into *E. coli* DH10B. These genes were originally selected from fecal samples (six of eight) and latrine samples (two of eight) from a peri-urban setting in Peru^26^ (Supplementary Data 1). We additionally include nine genes of environmental origin which were originally selected from agricultural soils in Michigan (five of nine) and grassland soils in Minnesota (four of nine)^11^ (Supplementary Data 1). Lastly, we included one homolog identified in the human pathogen *L. longbeacheae* (*tet*(56))^11^ and *tet*(X), which was originally discovered in the commensal *B. fragilis*, but has since been identified in numerous pathogens^9,29,30^. These genes were selected to encompass the representative phylogenetic diversity in the broader set and based on the availability of metagenomic DNA for subcloning. We performed antimicrobial susceptibility testing using broth microdilution for these recombinant constructs against 11 tetracycline compounds, including anhydrotetracycline. All computationally predicted tetracycline-inactivating enzymes tested had a bona fide resistance function when subcloned from their metagenomic source into *E. coli*. We found that resistance phenotypes, like genotypes, clustered according to habitat of origin (Fig. 2). Each of the eight human gut-derived genes displayed pan-tetracycline resistance. While all gut-derived tetracycline-inactivating enzymes conferred high-level resistance to tigecycline, minocycline, eravacycline, and omadacycline, soil-derived enzymes were all susceptible or intermediate to these drugs. Thus, resistance to latest-generation tetracyclines mediates phenotypic clustering between the soil-derived and gut-derived genes and discriminates between habitats of origin. Our functional identification of 69 diverse tetracycline inactivators from soil, human gut, animal gut, and latrine metagenomes indicate that tetracycline resistance by inactivation is widespread in diverse metagenomes^14,15^. This includes, in the case of genes identified from gut metagenomes, high-level resistance to latest-generation tetracyclines.

### Tet(X7) confers tetracycline resistance

Each of the eight gut-derived tetracycline-inactivating enzymes encoded resistance to every tetracycline currently approved for clinical use, including tigecycline, eravacycline, and omadacycline. Tigecycline was approved for human use in 2005, and is an antibiotic of last resort for infections, including those caused by carbapenem-resistant *Enterobacteriaceae*^17,31^. Eravacycline was approved in August 2018 for treatment of complex intra-abdominal infections, and omadacycline was approved in October 2018 for treatment of community acquired bacterial pneumonia and acute skin and skin structure infections^32,33^. Reports of resistance to eravacycline and omadacycline have been limited to date. Eravacycline resistance in *Klebsiella pneumoniae* has been attributed to overexpression of the OqxAB and MacAB efflux pumps^34^, and nonsusceptibility (MIC 4 µg/mL) has been observed in *Escherichia coli* DH10B heterologously overexpressing Tet(X). The recent reports of plasmid-encoded Tet(X3) and Tet(X4) in Enterobacteriaceae and *Acinetobacter* strains isolated from animal and human sources, encoding phenotypic resistance to eravacycline and omadacycline in both whole-cell assays and murine infection models, are of potential immediate clinical concern^14,15^. Approval of these drugs motivate further study of this mode of resistance. To better understand the mechanistic basis of resistance to latest-generation tetracyclines by these types of emerging resistance determinants, we selected a gut-derived gene, *tet(X7)*, for further analysis. We found that media conditioned by *E. coli* expressing Tet(X7) supported the growth of susceptible *E. coli*, indicating that the mechanism of resistance is consistent with drug inactivation.

**Fig. 3:**
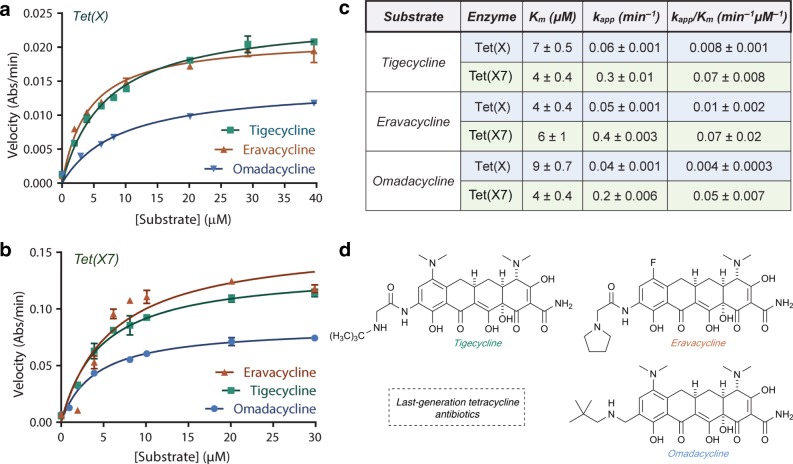
Enzymology of tetracycline inactivation by Tet(X7). Michaelis–Menten kinetics of tetracycline-inactivating enzyme-mediated degradation of last-generation tetracyclines. **a** Michaelis–Menten plot of Tet(X) degradation of tigecycline and eravacycline. **b** Michaelis–Menten plot of Tet(X7) degradation of last-generation tetracyclines. **c** Apparent *K*_m_, *k*_cat_, and catalytic efficiencies for the tetracycline-inactivating enzyme-mediated degradation of tigecycline, eravacycline, and omadacycline. **d** Last generation tetracycline antibiotics tigecycline, eravacycline, and omadacycline. Error bars represent standard deviation for three independent trials.

We characterized the in vitro degradation of tigecycline, eravacycline, or omadacycline by recombinant *N*-His_6_-tagged Tet(X7), indicated by a time- and enzyme-dependent decrease in the ~400-nm absorbance band in the optical absorbance spectrum that is associated with disruption of the conserved β-diketone chromophore found in all tetracyclines^11^ (Supplementary Fig. 1a). To understand substrate binding and catalytic efficiency for latest-generation tetracycline inactivation by Tet(X7), we measured apparent Michaelis–Menten kinetic parameters by continuously monitoring the decrease in optical absorbance at 400 nm under steady-state conditions (Fig. 3b). For comparison, we also determined Michaelis–Menten kinetic parameters of Tet(X)-mediated inactivation of tigecycline and eravacycline (Fig. 3a). The apparent catalytic efficiency of Tet(X7) was five times greater than that of Tet(X) for degradation of eravacycline (*k*_*cat*_*/K*_*M*_ values of 0.07 ± 0.02 and 0.01 ± 0.002 µM^−1 ^min^−1^, respectively) and eight times greater for degradation of tigecycline (*k*_*cat*_*/K*_*M*_ values of 0.07 ± 0.01 and 0.01 ± 0.001 µM^−1^ min^−1^, respectively; Fig. 3c, d). This difference in apparent catalytic efficiencies is largely mediated by increased substrate turnover by Tet(X7) compared with Tet(X), as *K*_*M*_ values are of similar magnitude across all pairwise enzyme-substrate combinations. Indeed, the apparent rate constants (*k*_*cat*_) for Tet(X7)-mediated degradation of tigecycline, eravacycline, and omadacycline are an order of magnitude greater than those observed for Tet(X) (Fig. 3c). We previously reported kinetic parameters for gut-derived Tet(X) and Tet(X7) (referred to as Tet(X)_3 in ref. ^23^) compared with soil-derived Tet(50) using first (tetracycline, chlortetracycline, and demeclocycline) and second (oxytetracycline) generation tetracycline antibiotics and observed that Tet(50) and Tet(X7) displayed similar catalytic efficiencies for inactivating first and second-generation tetracyclines that were ~fivefold greater than Tet(X). However, in contrast to Tet(X) and Tet(X7), the soil-derived Tet(50) was unable to inactivate tigecycline, a third-generation tetracycline (Fig. 2)^23^. Here, we demonstrate that Tet(X7) encodes the improved catalytic efficiency of the soil-derived enzyme Tet(50) and additionally has an expanded substrate scope for inactivation of third-generation tetracyclines.

When enzymatic reactions using Tet(X) and Tet(X7) were analyzed by liquid chromatography-mass spectrometry, we observed the primary product of omadacycline, tigecycline, and eravacycline degradation is consistent with monohydroxylation (Supplementary Fig. 1b, c). For each latest-generation tetracycline substrate, there was a time- and enzyme-dependent decrease in the relative extracted ion counts of tetracycline substrate (*m/z* for [M + H]^+^ equal to 586, 557, 559 for tigecycline, eravacycline, and omadacycline, respectively), and a corresponding increase in the relative extracted ion counts of monooxygenated product (*m/z* for [M + H]^+^ equal to 602, 573, 575 for tigecycline, eravacycline, and omadacycline, respectively; Supplementary Fig. 1b). Enzyme-dependent antibiotic degradation was also confirmed by HPLC with detection by optical absorbance (Supplementary Fig. 1c). Tet(X) has previously been shown to monohydroxylate the C11a-position of tetracyclines^13^. We show that the monohydroxylated product of Tet(X) and Tet(X7) reaction with tigecycline coelute (Supplementary Fig. 2), indicating that the site of hydroxylation by Tet(X7) is also C11a.

### Tet(X7) structurally resembles Tet(X)

**Fig. 4:**
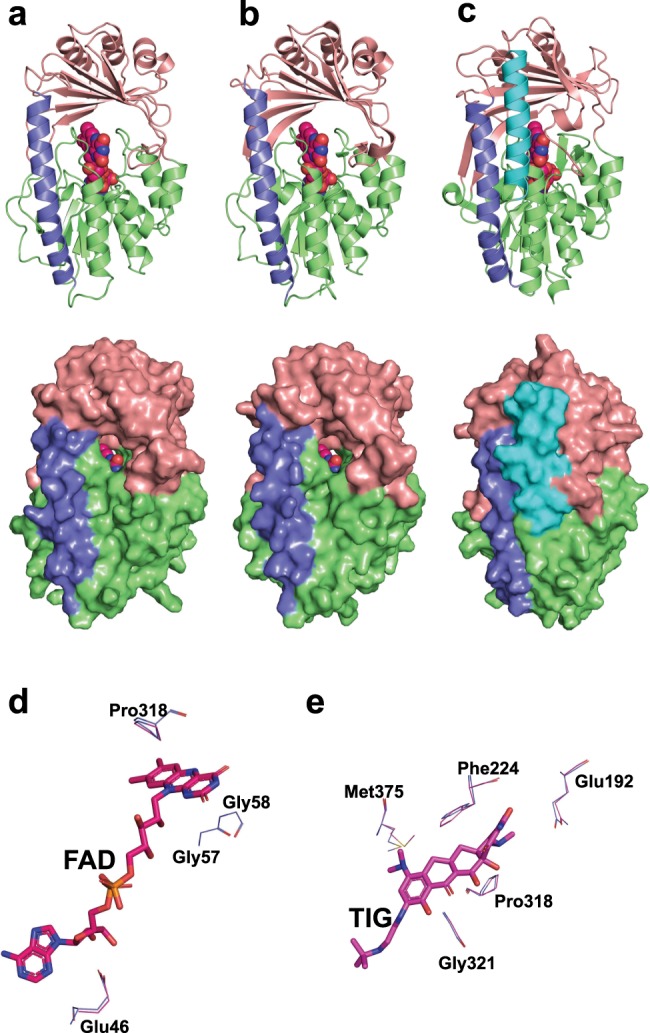
Conserved architecture of different tetracycline-inactivating enzymes. Crystal structures of Tet(X7) (**a**), Tet(X) (**b**), and Tet(50) (**c**). All three enzymes have a conserved FAD-binding Rossmann-fold (green), a substrate-binding domain (pink) and a bridge helix (purple). Tet(50) has an additional helix (cyan). The bound FAD is represented spherically. **d** The Tet(X7) structure is aligned to tigecycline (TIG)-bound Tet(X) structure (PDB ID: 4A6N). Nonbonded interactions of the bound FAD (stick) are conserved between Tet(X7) (blue) and Tet(X). **e** The nonbonded interactions of tigecycline (TIG) are also conserved between the two proteins.

We solved an X-ray crystal structure of Tet(X7) at a resolution of 2.55 Å (Table 1), and observed a similar architecture to previously reported tetracycline inactivators^12,35^ with an FAD-binding Rossmann-type fold domain, a tetracycline-binding domain, and a C-terminal α-helix that bridges the two domains (Fig. 4a–c). Structurally, the enzyme resembles Tet(X) more so than the tetracycline-inactivating enzymes Tet(50,51,55,56) owing to the presence of a single C-terminal bridging helix, rather than the two observed in the soil-derived enzymes. We examined the structural differences between Tet(X7) and Tet(X) that could explain the enhanced phenotypic resistance of Tet(X7) against tigecycline and minocycline, despite ~86% amino-acid identity. Structure alignment of Tet(X7) and Tet(X) (PDB ID: 2XDO, chain A) demonstrated a low overall root-mean-square deviation of 0.37 Å. The FAD in Tet(X7) retains an IN-conformation analogous to the substrate-free Tet(X) structure^36^. The FAD-binding residues (Glu46, Gly57, Gly58, Pro318) and substrate-binding residues (Gln192, Phe224, Pro318, Gly321, Met375) are conserved between the two proteins (Fig. 4d, e). Furthermore, multiple sequence alignment revealed that these residues are conserved between all Tet(X)-like FMOs.

**Table 1:** Data collection and processing statistics for Tet(X7).

	Crystal 1 name
*Data collection*	
Space group	*P* 2_1_2_1_2_1_
Cell dimensions	
* a*, *b*, *c* (Å)	57.130, 131.970, 136.970
a, b, g (°)	90.00, 90.00, 90.00
Resolution (Å)	19.77–2.55 (2.641–2.55)
Rmeas	0.09138 (0.8967)
I/sI	17.31 (2.26)
Completeness (%)	99.50 (99.74)
Redundancy	6.9 (6.5)
*Refinement*	
Resolution (Å)	19.77–2.55
No. of reflections	34,427
Rwork/Rfree	0.2070/0.2436
No. of atoms	
Protein	5800
Ligand/ion	106
Water	62
B-factors	
Protein	63.82
Ligand/ion	49.63
Water	49.27
R.m.s. deviations	
Bond lengths (Å)	0.004
Bond angles (°)	0.72

Mapping of divergent residues on a structural alignment of Tet(X7) and Tet(X) structures revealed that these residues are peripheral and are far removed from the FAD- and substrate-binding regions (Supplementary Fig. 3). Therefore, it is unlikely that the altered residues directly play a role in substrate-binding or catalysis. Instead, it is plausible that these differences allow for altered conformational dynamics and may allosterically affect enzyme activity. This is consistent with prior studies using directed evolution that identified several mutations in Tet(X) outside of the active site that enhance Tet(X) enzyme activity against tigecycline^22^. Many differences map to the outer surface of the bridge-helix. The changes exhibited a pattern where, in Tet(X7), the substituted amino acids were either smaller (Lys351Glu, Ile359Ala, Glu366Ala, and Lys377Ser) or were less hydrophilic (Glu366Ala, Thr369Ile, Gln370Ile; Supplementary Fig. 4). We suspect that these differences may affect the dynamics of the hydrophobic cavity resulting in faster enzyme turnover. A further difference that may play a role in substrate tolerance is a ten-amino acid C-terminal truncation of Tet(X7) compared with Tet(X) and Tet(X3) (seven amino acid difference from Tet(X4); Supplementary Fig. 4). We have previously described the role of C-terminal helices in governing substrate specificity in tetracycline-inactivating FMOs^12^, and speculate that the substitutions in the C-terminal helix of Tet(X7) may similarly mediate the more efficient inactivation of next-generation tetracyclines by Tet(X7) compared with Tet(X) (Fig. 3c).

### Tet(X7) is functional in a clinical *P. aeruginosa* isolate

While functional metagenomics is a useful method for identifying resistance genes in a sequence- and culture-unbiased manner, it is poorly equipped to associate specific resistance determinants with their native hosts. Thus, we sought to identify homologs of our set of functionally validated resistance genes in sequenced clinical isolates. We identified Pa-3, a *Pseudomonas aeruginosa* strain isolated from a 45-year-old male cystic fibrosis patient’s tracheal aspirate in a tertiary care hospital ICU in Pakistan in December 2016. This strain was predicted to encode an FAD-dependent oxidoreductase homolog by BLAST^37^, which we identified as having 100% nucleotide identity to Tet(X7). We conducted antimicrobial susceptibility testing on this strain using disk diffusion for a panel of clinical antibiotics. Pa-3 was resistant to all antibiotics tested with the exception of piperacillin/tazobactam, a β-lactam/β-lactamase inhibitor combination, and colistin (Table 2). *P. aeruginosa* is a serious clinical threat^38^, and acquisition of resistance to latest-generation tetracyclines further exacerbates this hazard.

**Table 2:** Zone of clearance (mm) and phenotypic resistance determination for clinical *Pseudomonas aeruginosa* isolates.

	Pa-3		Pa-8		ATCC 27853
Antibiotic	Kirby–Bauer (mm)	Interpretation	Kirby–Bauer (mm)	Interpretation	Kirby–Bauer (mm)
Delafloxacin	6	Resistant	6	Resistant	23–29
Ceftazidime	6	Resistant	6	Resistant	22–29
Cefepime	6	Resistant	6	Resistant	25–31
Meropenem	6	Resistant	6	Resistant	27–33
Imipenem	6	Resistant	25	Sensitive	20–28
Piperacillin/tazobactam	24	Sensitive	19	Intermediate	25–33
Ceftolozane/tazobactam	6	Resistant	29	Sensitive	25–31
Ceftazidime/avibactam	6	Resistant	26	Sensitive	25–31
Ciprofloxacin	6	Resistant	6	Resistant	25–33
Levofloxacin	6	Resistant	6	Resistant	19–26
Gentamicin	6	Resistant	6	Resistant	17–23
Amikacin	6	Resistant	17	Sensitive	18–26
Trimethoprim/sulfamethoxazole	6	NA	6	NA	—
Fosfomicin	12	Resistant	15	Intermediate	—
Colistin	13	Sensitive	15	Sensitive	11–17
Aztreonam	17	Intermediate	16	Intermediate	23–29
Doxycycline	6	NA	6	NA	—
Minocycline	6	NA	6	NA	—
Tigecycline	6	NA	6	NA	9–13
Nitrofurantoin	6	NA	6	NA	—
Carbapenem inactivation assay	14	Positive	27	Positive	—

NA indicates that no interpretive criteria have been established. ATCC 27853 values are disk diffusion QC ranges provided by the CLSI^63^.

In addition to antimicrobial susceptibility testing by disk diffusion, we performed broth microdilution for a subset of tetracycline antibiotics for which disks are not yet commercially available, against Pa-3 (the strain encoding Tet(X7)), Pa-8 (another clinical isolate from the same collection from Pakistan which did not encode Tet(X7)), and the *P. aeruginosa* type strain ATCC 27853 (also lacking Tet(X7)). Each *P. aeruginosa* isolate displayed nonsusceptibility to tigecycline, eravacycline, and omadacycline, even in the absence of a putative tetracycline inactivator (Table 3). This is likely due to drug efflux, as low-level OprM mediated resistance to tigecycline in *P. aeruginosa* PAO1 has been previously reported^39,40^. Although tigecycline, eravacycline, and omadacycline breakpoints do not yet exist for *P. aeruginosa*, Pa-3 was four- to eightfold more non-susceptible to these drugs than Pa-8 and ATCC 27853. Our results with *P. aeruginosa* expand the repertoire of urgent-threat MDR clinical pathogens with demonstrated nonsusceptibility to either eravacycline or omadacycline via enzymatic inactivation^14,15^. We anticipate clinical deployment of these antibiotics will lead to selection for and expansion of this mode of resistance in both hospital and community settings.

**Table 3:** Anhydrodemeclocycline at subinhibitory concentrations can partially rescue tetracycline, tigecycline, eravacycline, and omadacycline efficacy against *Pseudomonas aeruginosa* Pa-3, *E. coli* DH10B heterologously expressing Tet(X7), *Pseudomonas aeruginosa* ATCC 27853, and *E. coli* DH10B with an empty pZE21 vector.

	*Pseudomonas aeruginosa* Pa-3		*Escherichia coli* DH10β + *tet(X7)*		*Pseudomonas aeruginosa* ATCC 27853		*Escherichia coli* DH10β + *pZE21*	
Antibiotic	MIC- 0 µg/mL aDem	MIC- 8 µg/mL aDem	MIC- 0 µg/mL aDem	MIC- 8 µg/mL aDem	MIC- 0 µg/mL aDem	MIC- 8 µg/mL aDem	MIC- 0 µg/mL aDem	MIC- 8 µg/mL aDem
Tetracycline	128	32	256	128	32	32	8	4
Tigecycline	64	64	32	2	8	8	1	1
Eravacycline	64	32	8	8	16	16	1	1
Omadacycline	256	256	64	8	128	128	1	1

In total, 8 µg/ml anhydrodemeclocycline is equivalent to 18 µM anhydrodemeclocycline.

Media conditioned by Pa-3 supported the growth of susceptible *E. coli*, while media conditioned by 27853 or Pa-8 could not, confirming that the mechanism of resistance in Pa-3, but not Pa-8 or 27853, involves antibiotic inactivation. Clinical use of latest-generation tetracyclines may select for dissemination of this strain. *Pseudomonas spp*. have highly plastic genomes and undergo horizontal gene transfer at rates greater than observed for other genera^11,12^, so even chromosomally encoded genes are possible threats for transfer beyond this strain. Lastly, we observed that Tet(X7) was syntenic with putative rolling circle transposases (Pfam:PF04986), suggesting that they may be poised for horizontal transfer^2,41^.

### Anhydrotetracycline analogues rescue tetracycline efficacy

**Fig. 5:**
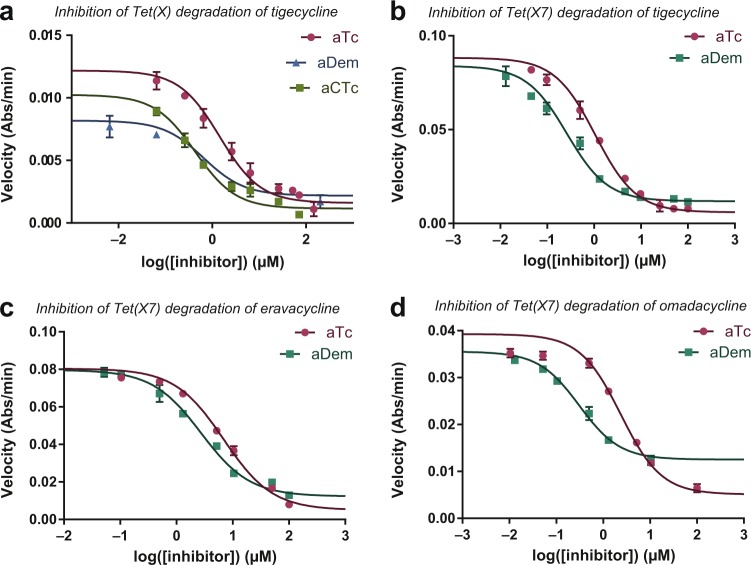
Anhydrotetracycline and analogues inhibit enzymatic inactivation of latest-generation tetracyclines by Tet(X7). **a** In vitro, anhydrotetracycline, anhydrochlortetracycline, and anhydrodemeclocycline inhibit Tet(X) modification of tigecycline. Likewise, anhydrotetracycline and anhydrodemeclocycline inhibit in vitro enzymatic modification of tigecycline (**b**), eravacycline (**c**), and omadacycline (**d**) by Tet(X7). Error bars represent standard deviation for three independent trials.

We have previously shown that anhydrotetracycline is an inhibitor of tetracycline-inactivating enzymes in vitro and that this inhibition is sufficient to rescue tetracycline efficacy against *E. coli* strains heterologously expressing tetracycline-inactivating enzyme^12,23^. We reasoned that this strategy might likewise prevent degradation of latest-generation tetracyclines by Tet(X7). To this end, we evaluated in vitro inhibitory activity of anhydrotetracycline and anhydrodemeclocycline against the Tet(X7)-mediated degradation of tigecycline, eravacycline, and omadacycline. Apparent half-maximal inhibitory concentrations (IC_50_s) showed that anhydrotetracycline potently inhibited Tet(X7) degradation of tigecycline, eravacycline, and omadacycline (IC_50_s of 1.06 ± 0.108 µM, 6.89 ± 0.655 µM, and 2.37 ± 0.510 µM, respectively). Moreover, anhydrodemeclocycline also potently inhibited the Tet(X7) degradation of these latest-generation substrates, with IC_50_s of 0.26 ± 0.04 µM, 2.75 ± 0.26 µM, and 0.31 ± 0.04 µM, respectively (Fig. 5a–d). We reason, based on the structural similarity to previously published cocrystal structures of Tet(50) (PDB ID: 5TUF**)** with anhydrotetracycline, that the mechanism of inhibition likely occurs via mixed competitive and noncompetitive inhibition^12^.

In order to extend our in vitro studies, we sought to determine whether anhydrotetracycline or anhydrodemeclocycline could restore the activity of latest-generation tetracyclines against Pa-3, the clinical *P. aeuruginosa* isolate that encodes Tet(X7). Anhydrotetracycline at 32 µg/mL caused a two- to fourfold increase in sensitivity of Pa-3 to tigecycline, eravacycline, or omadacycline (Table 3). In the same way, 8 µg/mL anhydrodemeclocycline was sufficient to cause a fourfold increase in sensitivity to tetracycline, and a twofold increase in sensitivity to tigecycline, eravacycline, or omadacycline (Table 3). Anydrodemeclocycline exhibits an MIC of 16 µg/mL against both *E. coli* + Tet(X7) and *P. aeruginos*a Pa-3 (Table 3). While anhydrodemeclocycline does exhibit antibiotic activity, we test for inhibition at concentrations below the minimum inhibitory concentration. We believe that the antibiotic activity of anhydrodemeclocycline is due to targeting of the cell membrane rather than inhibition of the 30S ribosomal subunit, based on prior investigations of anhydrotetracycline antibiotic activity^42^. Our data indicate that the activity exhibited by anhydrodemeclocycline in our assay is due to inhibition of Tet(X7) rather than additive antibiotic activity. Thus, this adjuvant strategy has promise for restoring the efficacy of tetracycline antibiotics, including latest-generation tetracyclines, against bacteria that resist tetracycline through inactivation.

## Discussion

Since their discovery from extracts of *Streptomyces aureofaciens* in 1948, the tetracyclines have become one of the most widely used classes of antibiotics in agriculture, aquaculture, and the clinic due to their broad antimicrobial spectrum, oral availability, and low cost^8,43^. Extensive use over the past seven decades has selected for the expansion of tetracycline resistance in environmental microorganisms^44^, human and animal commensals^45^, and among bacterial pathogens^46^. Tetracycline use is particularly prevalent in agriculture, with tetracyclines comprising 66% of total therapeutic antibiotic use in livestock^47^. Widespread anthropogenic use has resulted in detectable ng/µL to µg/L quantities of tetracyclines in livestock manure and wastewater^48^, with tetracycline concentration directly correlating with changes in microbial community composition and increase in antibiotic resistance^49^. As a result of widespread anthropogenic use, tetracycline resistance is now widespread.

The rise in tetracycline resistance has been partially countered by the development of fully synthetic (eravacycline) and semisynthetic (tigecycline) latest-generation tetracyclines^8^. The first-generation natural product antibiotics, including chlortetracycline (1948), oxytetracycline (1951), and tetracycline (1953), were followed by the semisynthetic second-generation tetracyclines, including doxycycline (1967) and minocycline (1971), and latest-generation tetracyclines, such as tigecycline (2005), eravacycline (2018) and omadacycline (2018)^50^. Meeting clinical needs and overcoming established resistance mechanisms, such as efflux and ribosome protection, has motivated each new wave of tetracycline development. As a result, the tetracyclines are still widely used in agriculture and medicine^51^, and remain viable therapies for a range of indications^43,52^. The emergence of Tet(X)^29^, Tet(X3)^14^, Tet(X4)^14,15^, and now Tet(X7) in clinical pathogens now establishes covalent inactivation of tetracycline antibiotics as a bona fide clinical resistance mechanism. It is unclear if modification of the tetracycline scaffold will be sufficient to keep pace with the emergence of genes encoding tetracycline inactivation on the global scale.

Until recently, Tet(X) was the only known enzyme capable of providing both tetracycline and tigecycline resistance, first identified in nonpathogenic bacteria^53^. In 2013, *tet(X)* was discovered to be present and functional in human pathogens. Eleven isolates from the Enterobacteriaceae, Comamonadaceae, and Pseudomonadaceae families from clinical urine specimens in a hospital in Sierra Leone were found to encode *tet(X)*^29^. This resistance determinant has now been reported in four out of the six ESKAPE pathogens (*Enterococcus faecium, Staphylococcus aureus, Klebsiella pneumoniae, Acinetobacter baumannii, Pseudomonas aeruginosa*, and *Enterobacter species)*, which are the leading causes of hospital-acquired infections around the world^29,30^. The discovery of a gene encoding tetracycline inactivation in nosocomial pathogens foreshadowed the increasing clinical resistance to latest-generation tetracycline antibiotics, including the discovery of plasmid-encoded *tet(X3)* and *tet(X4)* in MDR Enterobacteriaceae and *Acinetobacter* spp^14,15^, along with chromosomal *tet(X7)* in *Pseudomonas aeruginosa* (this work). It is now clear that future dependence on new tetracycline antibiotics including tigecycline, eravacycline, and omadacycline to treat infections caused by MDR Gram-negative pathogens may be compromised by widespread tetracycline-inactivating enzymes. Expanded global use of last-generation tetracyclines in clinical and agricultural settings will increase selective pressure for tetracycline-inactivating genes and promote dissemination.

Prospecting for environmental and clinical *tet(X)* homologous genes is a promising method to proactively assess the landscape of tetracycline resistance via chemical inactivation. Our work has now revealed that tetracycline inactivation occurs widely across environmental, human commensal, and pathogenic microbes. The *tet(X)* gene originally discovered in human commensal *Bacteroides fragilis* has been acquired by human pathogenic Gram-negative bacteria^29^. The related tetracycline-inactivating enzymes are widespread in soil environments and confer resistance to natural and early-generation semisynthetic tetracyclines^11^. The clustering of tetracycline-inactivating enzymes with microbial habitat of origin and resistance phenotype that we show here parallels pharmaceutical development and clinical use of tetracycline antibiotics. Since FAD-dependent oxidoreductases are ubiquitous, and there is low sequence similarity between the tetracycline-inactivating enzymes and *tet(X)* family of genes, functional selection is critical to properly survey the resistance landscape. Expanding the pool of functionally similar resistance determinants is the first step in deconvoluting the evolutionary link between soil tetracycline-inactivating enzymes and Tet(X) homologs. Further structural and biochemical characterization of tetracycline-inactivating enzyme sub-classes is critical for understanding the molecular basis for antibiotic inactivation and finding functional connections in sequence evolution that will feed predictive models. For now, Tet(X) homologs appear to be a more likely clinical threat than the soil tetracycline-inactivating enzymes, but ignorance to the full reservoir of tetracycline-inactivating genes blinds clinicians and drug developers to the most urgent needs in therapeutic advances. The observance of human-associated genes which confer resistance to next-generation tetracyclines likely reflects the promiscuous nature of the FMO family, which is highly adaptable to a wide range of substrates. Specific selection for tetracycline substrates in human versus environmental habitats most likely arose from natural production of these types of tetracycline scaffolds in their respective microbial communities, since at least the human-associated enzymes clearly existed before the latest-generation drugs were clinically deployed. Directed evolution can be useful to fill some of the gaps in these known unknowns, as has been demonstrated for other clinically important resistance determinants (e.g., beta-lactamases)^54^. An ideal goal is to predict phenotypic resistance by sequence alone and employ preemptive strategies for managing resistance early in development instead of waiting for an increase in clinical resistance events^55^.

Better understanding the molecular recognition of tetracycline substrate recognition by tetracycline-inactivating enzymes can help design the next generation of structurally modified tetracyclines that can stealthily evade inactivation or maintain efficacy after a catalyzed oxidation event. More structural information dictating enzyme function and substrate recognition, such as the 2.55 Å resolution structure of Tet(X7) reported here, is needed to guide the rational design of next-generation tetracyclines capable of evading enzymatic inactivation. Recent advancements in the total chemical synthesis of tetracyclines, such as eravacycline^56^, can play a critical role in providing synthetic access to rationally designed compounds. Structure-based methods can also play a key role in designed inhibitors for use in combination therapies with tetracycline antibiotics^23^. We provide evidence that anhydrotetracycline and analogs can inhibit broad-spectrum tetracycline-inactivating enzymes, including Tet(X) and Tet(X7) present in clinical pathogens, to rescue tetracycline activity in whole cells. Combination therapy appears to be the gold standard for managing resistance by antibiotic inactivation as supported by the steady development of β-lactam/β-lactamase inhibitor combinations^57^. It is crucial to invest in the development of combination therapies before the isolated use of last-generation tetracyclines as stand-alone agents becomes functionally obsolete. This is of particular import given a recent report that *tet(X3)* compromises tigecycline efficacy in a mouse model of *Acinetobacter baumanii* thigh infection^14^. Our data suggest that further exploration of the anhydrotetracycline scaffold as a source of novel inhibitors to rescue tetracycline activity in the face of widespread tetracycline resistance by inactivation is warranted^23^.

While the environmental resistome has been shown to be extensive, unambiguous links between the environmental and clinical resistomes have remained elusive. Here, we explicitly demonstrate overlap between microbial habitats by identifying a common family of resistance genes encoding tetracycline-inactivating enzymes present in environmental, commensal, and pathogenic bacterial hosts. The results presented herein motivate continued surveillance of tetracycline resistance by inactivation in environmental and clinical settings to better understand the origins, evolution, and dissemination of resistance. This work underscores the value in proactive screening of antimicrobial agents in the pipeline for resistance determinants present in diverse microbial communities such that strategies to minimize their eventual clinical impact can be explored early in the development pipeline.

## Methods

### Identification of candidate tetracycline inactivators in sequenced functional selections

In order to identify additional tetracycline inactivators, we queried previously sequenced functional selections of human gut, animal gut, latrine, and soil metagenomes^2,3,25–28^ [GenBank accession numbers: JX009202-JX009380, KJ691878-KJ696532, KU605810-KU608292, KF626669-KF630360, KX125104-KX128902, KU543693-KU549046]. While the functional metagenomic protocol is described in detail in the papers accompanying these depositions, we briefly describe the method below:

*Functional metagenomic library creation*: Functional metagenomic libraries were constructed as previously described^25,58^. Approximately 5 µg of purified extracted total metagenomic DNA was used as a starting material for metagenomic library construction. To create small-insert metagenomic libraries, DNA was sheared to a target size of 3000 bp using the Covaris E210 sonicator following the manufacturer’s recommended settings. Sheared DNA was concentrated by QIAquick PCR Purification Kit (Qiagen) and eluted in 30 μl nuclease-free H_2_O. Then the purified DNA was size-selected on an agarose gel to a range of 1000–6000-bp DNA fragment through a premade 0.75% Pippin gel cassette. Size-selected DNA was then end-repaired using the End-It DNA End Repair kit (Epicenter). End-repaired DNA was then purified using the QIAquick PCR purification kit (Qiagen), and quantified using the Qubit fluorometer BR assay kit (Life Technologies) and ligated into the pZE21-MCS-1 vector at the HincII site. The pZE21 vector was linearized at the HINCII site using inverse PCR with PFX DNA polymerase (Life Technologies). End-repaired metagenomic DNA and linearized vector were ligated together using the Fast-Link Ligation Kit (Epicenter) at a 5:1 ratio of insert:vector. After heat inactivation, ligation reactions were dialyzed for 30 min using a 0.025-µm cellulose membrane (Millipore catalog number VSWP09025), and the full reaction volume used for transformation by electroporation into 25 μl *E. coli* MegaX (Invitrogen) according to the manufacturer’s recommended protocols. Cells were recovered in 1 ml recovery medium (Invitrogen) at 37 °C for 1 h. Recovered cells were inoculated into 50 ml of LB containing 50 μg/ml kanamycin, and grown overnight. The overnight culture was frozen with 15% glycerol and stored at −80 °C for subsequent screening.

*Antibiotic selection of functional metagenomic libraries:* Each metagenomic library was selected for resistance at a concentration of 8 μg/ml tetracycline or 2 μg/ml tigecycline plus 50 μg/ml kanamycin for plasmid library maintenance on Meuller Hinton agar. After plating (using sterile glass beads), antibiotic selections were incubated at 37 °C for 18 h to allow the growth of clones containing an antibiotic resistance-conferring DNA insert. After overnight growth, all colonies from a single antibiotic plate (library by antibiotic selection) were collected by adding 750 μl of 15% MH-glycerol to the plate and scraping with an L-shaped cell scraper to gently remove colonies from the agar. The bacterial cells were then stored at −80 °C before PCR amplification of antibiotic-resistant metagenomic fragments and Illumina library preparation.

*Functional metagenomic library sequencing:* Cells were lysed by freezing, and the resulting supernatant was used as a template for amplification of resistance-conferring DNA fragments by PCR with Taq DNA polymerase (New England BioLabs). The amplified metagenomic inserts were then cleaned using the Qiagen QIAquick PCR purification kit, and quantified using the Qubit fluorometer HS assay kit (Life Technologies).

For amplified metagenomic inserts from each antibiotic selection, elution buffer was added to the PCR template for a final volume of 200 μl, and sonicated in a half-skirted 96-well plate on a Covaris E210 sonicator with the following setting: duty cycle, 10%; intensity, 5; cycles per burst, 200; sonication time, 600 s. Following sonication, sheared DNA was purified and concentrated using the MinElute PCR Purification kit (Qiagen), and eluted in 20 μl of pre-warmed nuclease-free H_2_O. In the first step of library preparation, purified sheared DNA was end-repaired. Next, to each end-repaired sample, 5 μl of 1 μM pre-annealed, barcoded sequencing adapters were added. After the addition of barcoded adapters, samples were incubated at 16 °C for 40 min, and then for 10 min at 65 °C. The pooled, adaptor-ligated, sheared DNA was then size-selected to a target range of 300–400 bp on a 2% agarose gel in 0.5X TBE, stained with GelGreen dye (Biotium) and extracted using a MinElute Gel Extraction Kit (Qiagen). The purified DNA was amplified and quantified using the Qubit fluorometer HS assay kit (Life Technologies), and 10 nM of each sample was pooled for sequencing. Subsequently, samples were submitted for paired-end 101-bp sequencing using the Illumina NextSeq platform.

*Assembly and annotation of functionally selected contigs:* Illumina paired-end sequence reads were binned by barcode (exact match required), such that independent selections were assembled and annotated in parallel. Assembly of the resistance-conferring DNA fragments from each selection was achieved using PARFuMS^2^ (parallel annotation and reassembly of functional metagenomic selections), a tool developed specifically for the high-throughput assembly and annotation of functional metagenomic selections.

Open-reading frames (ORFs) were predicted in assembled contigs using MetaGeneMark^8^ and annotated by searching amino-acid sequences against Pfam, TIGRfam, and an ARG specific profile hidden Markov model (pHMM) database, Resfams^24^ (http://www.dantaslab.org/resfams), with HMMER3^10^. MetaGeneMark was run using default gene-finding parameters while *hmmscan* (HMMER3) was run with the option --*cut*_*ga* as implemented in the script *annotate_functional*_*selections*.*py*.

The majority of functionally identified tetracycline resistance genes are efflux pumps or ribosomal protection proteins. To identify resistance determinants which might function by monooxygenation of the tetracycline scaffold, annotations were queried for putative tetracycline-inactivating function based on string matches to one of the following keywords in Pfam and TIGRfam annotations: “FAD dependent oxidoreductase”, “oxidoreductase”, “FAD binding domain”.

### Construction of maximum likelihood phylogenies

A multiple sequence alignment of predicted tetracycline-inactivating enzymes was created with MAFFT using the L-INS-i method^59^. A maximum likelihood phylogeny was then constructed using RAxML with GAMMA model of rate heterogeneity and JTT empirical base frequencies with 100 bootstraps^60^. Trees were visualized and decorated with metadata using iTol^61^. Multiple sequence alignments were visualized with MView^62^.

### Subcloning predicted tetracycline inactivators from metagenomic source

Specific predicted tetracycline inactivators were selected for subcloning for downstream phenotypic analysis if they met each of the following criteria: (1) the open-reading frame contained a start codon; (2) the open-reading frame contained a stop codon; and (3) the open-reading frame was greater than 1000 base pairs, but less than 1500 base pairs. *tet(47-56)* and *tet(X)* had previously been subcloned^11^. Additional predicted tetracycline inactivators were amplified from their initial metagenomic source using Phusion Hi-Fidelity Polymerase (ThermoFisher) and primers as specified in Supplementary Table 1. Amplicons were ligated into pZE21 at the KpnI/HindIII sites using Fast-Link DNA Ligase (Lucigen) and transformed into *E. coli* MegaX DH10B cells (Invitrogen) via electroporation. The orientation and sequence of all inserts was confirmed by Sanger sequencing prior to phenotypic analyses.

### Antimicrobial susceptibility testing by microbroth dilution

Antibiotic susceptibility testing was performed in *E. coli* MegaX cells (Invitrogen) bearing the pZE21 expression vector with the tetracycline-inactivating gene of interest as previously described^12^. Minimum inhibitory concentrations (MICs) were determined according to Clinical and Laboratory Standards Institute (CLSI) procedures^63^ using Mueller–Hinton broth with 50 µg/mL kanamycin and a range of tetracycline antibiotic concentrations profiled via absorbance measurements at 600 nm (OD_600_) at 45-min intervals using the Synergy H1 microplate reader (Biotek Instruments, Inc) for 48 h at 37 °C and scored by eye following 20 h of growth at 37 °C.

### Pseudomonas aeruginosa isolation

*Pseudomonas aeruginosa* strain Pa-3 was isolated from tracheal secretions of a 45-year-old male patient admitted to intensive care unit at a tertiary care hospital in Pakistan in December 2016. A 100 µl suspension of the sample was plated initially on Blood Agar (Oxoid) and MacConkey Agar for 18–24 h at 37 °C followed by sub-streaking of morphologically distinct colonies on *Pseudomonas* cetrimide agar (Oxoid) incubated for 18–24 h at 37 °C. The isolate was identified using VITEK matrix-assisted laser desorption ionization–time of flight mass spectrometry (MALDI-TOF MS) with library version v2.3.3 (bioMérieux, Durham, NC). Following confirmation, the isolate was grown overnight in 1 mL TSB at 37 °C with shaking and stocked at −80 °C in 15% glycerol.

### *Pseudomonas aeruginosa* genomic DNA isolation

A suspension of ∼10 colonies from a blood agar plate was used for genomic DNA isolation with the QIAamp BiOstic Bacteremia DNA Isolation Kit (Qiagen) following the manufacturer’s protocols. Genomic DNA was quantified using a Qubit fluorometer dsDNA BR Assay (Invitrogen) and stored at −20 °C.

### *Pseudomonas aeruginosa* isolate sequencing library preparation

Genomic DNA was diluted to a concentration of 0.5 ng/µL prior to sequencing library preparation. Libraries were prepared using a Nextera DNA Library Prep Kit (Illumina) following the modifications described in Baym et al.^64^. Libraries were purified using the Agencourt AMPure XP system (Beckman Coulter), and quantified using the Quant-iT PicoGreen dsDNA assay (Invitrogen). Samples were submitted for 2 × 150 bp paired-end sequencing on an Illumina NextSeq High-Output platform at the Center for Genome Sciences and Systems Biology at Washington University in St. Louis with a target sequencing depth of 1 million reads per sample.

### Assembly and annotation of *Psuedomonas aeruginosa* genome

Genomes were assembled as previously described^58^. Prior to all downstream analysis, Illumina paired-end reads were binned by index sequence. Adapter and index sequences were trimmed using Trimmomatic v0.36^65^ using the following parameters: *java -Xms2048m -Xmx2048m -jar trimmomatic-0.33.jar PE -phred33 ILLUMINACLIP: NexteraPE-PE.fa:2:30:10:1:true*. Contaminating human reads were removed using DeconSeq^66^ and unpaired reads were discarded. Reads were assembled using SPAdes^67^ with the following parameters: *spades.py -k 21,33,55,77 –careful*. Contigs less than 500 bp were excluded from further analysis. Assembly quality was assessed using QUAST^68^. Genomes were annotated using Prokka^69^ with default parameters.

### Antimicrobial susceptibility testing for *Pseudomonas aeruginosa*

Susceptibility testing was performed using the Kirby–Bauer disk diffusion method on Mueller–Hinton agar (Hardy Diagnostics) in accordance with CLSI standards^63^. *Pseudomonas aeruginosa* ATCC 27853 was used as a quality control.

### Cloning, expression and purification of tetracycline-inactivating enzymes

All genes encoding tetracycline-inactivating enzymes were cloned into the pET28b(+) vector (Novagen) at BamHI and NdeI restriction sites. Constructs were transformed into BL21-Star (DE3) competent cells (Life Technologies). Cells harboring the plasmid were grown at 37 °C in LB medium containing a final concentration of 0.03 mg/mL kanamycin. Once cells reached an OD_600_ of 0.6, cells were cooled to 15 °C, and induced with 1 mM IPTG overnight. After this period, cells were harvested by centrifugation at 4000 rpm for 10 min at 4 °C. Cell pellets were suspended in 10 mL of 50 mM Tris (pH 8.0), 100 mM NaCl, 10 mM imidazole (pH 8.0), 1 mM PMSF, and 5 mM BME per 1 liter of LB medium and stored at −80 °C.

Cells were lysed by mechanical disruption using an Emulsiflex C5. The cell extract was obtained by centrifugation at 13,000 rpm for 30 min at 4 °C, and was applied onto nickel rapid run agarose beads (Goldbio) equilibrated with wash buffer (50 mM Tris (pH 8.0), 150 mM NaCl, 20 mM imidazole (pH 8.0), and 5 mM BME). The wash buffer was used to wash the nickel column three times with five column volumes. After washing, protein was eluted with five column volumes of elution buffer (wash buffer with 300 mM imidazole). The protein sample was further purified by gel chromatography using a HiLoad 16/600 Superdex 200 pg column (GE Healthcare) equilibrated with 10 mM Tris (pH 8.0), 150 mM NaCl, 5 mM dithioerythritol (DTT). The fractions containing the protein of interest were pooled and concentrated using a 30 K MWCO Amicon centrifugal filter (Millipore).

### Crystallization, data collection, and structure refinement

Tet(X7) was concentrated to 20 mg/mL, and crystallized by hanging drop vapor diffusion at 18 °C in 0.2 M ammonium sulfate and 20% (w/v) PEG 4000. Crystals were transferred to 20% ammonium sulfate, 20% (w/v) PEG 4000, and 20% glycerol for 15–30 s and flash-cooled in the liquid nitrogen. Diffraction data were collected at 100 K on beamline 4.2.2 (Advanced Light Source, Lawrence Berkeley National Laboratory in Berkeley, California). All data processing and structure analysis were performed using SBGrid^70^. Diffraction data was reduced and scaled using XDS^71^. Tet(X7) structure was solved by molecular replacement using Phaser^72^ with the substrate-free Tet(X) structure (PDB ID: 2XDO; percentage identity: ~86%) as a starting model. Structure refinement was performed in Phenix^73^ and Coot^74^. The final model was validated using the Molprobity server^75^.

### In vitro tetracycline-inactivation assays

In vitro reactions were performed as previously described^12^. Reactions were prepared in 100 mM TAPS buffer with 28.0 µM substrate, 0.24 µM enzyme, and an NADPH regenerating system consisting of the following components (final concentrations): glucose-6-phosphate (40 mM), NADP^+^ (4 mM), MgCl_2_ (1 mM), and glucose-6-phosphate dehydrogenase (4 U/ml). The regeneration system was incubated at 37 °C for 30 min to generate NADPH before use in reactions. Reactions were sampled at various time-points over a 2-h period, and 150-µL samples were transferred from the reaction vessel and quenched in four volumes of an acidic quencher consisting of equal parts of acetonitrile and 0.25 M HCl.

Products generated from the Tet(X7) enzymatic inactivation of the last-generation tetracyclines were separated by reverse-phase HPLC using a Phenomenex Luna C18 column (5 µm, 110 Å, 2×50 mm) and 0.1% trifluoroacetic acid in water (A) and acetonitrile (B) as mobile phase with optical absorbance detection at 260 nm. Injections of 10 µL sample volume were eluted using a linear gradient from 25% B to 75% B over 14 min at a flow rate of 1 mL/min. These samples were concurrently analyzed LCMS (reverse-phase HPLC) using an Agilent 6130 single- quadrupole instrument with G1313 autosampler, G1315 diode array detector, and 1200 series solvent module and separated using a Phenomenex Gemini C18 column, 50 × 2 mm (5 μm) with guard column cassette and a linear gradient of 0% acetonitrile + 0.1% formic acid to 95% acetonitrile + 0.1% formic acid over 20 min at a flow rate of 0.5 ml/min before analysis by electrospray ionization (ESI+).

### Kinetic characterization of tetracycline inactivation

Reactions were prepared in 100 mM TAPS buffer at pH 8.5 with 0–40 µM substrate, 504 µM NADPH, 5.04 mM MgCl_2_, and 0.4 µM enzyme. UV-visible spectroscopy measurements were taken in duplicate at 400-nm wavelength light with a Cary 60 UV/Vis system (Agilent) for 4 min at room temperature. Initial reaction velocities were determined by linear regression over the linear range of the reaction using the Agilent Cary WinUV Software, plotted against the concentration of the substrate, and fitted to the Michaelis–Menten equation using Graphpad Prism 6.

### In vitro characterization of anhydrotetracycline inhibition

IC_50_ values were determined for Tet(X7) by measuring the initial velocity of tetracycline degradation in the presence of varying concentrations of anhydrotetracycline (Adipogen Corporation, San Diego, CA) or anhydrodemeclocycline (prepared as described in ref. ^23^). The concentrations of tetracycline and NADPH were kept constant at 25 μM and 504 μM, respectively. Assays were prepared by combining all components except for enzyme and mixing manually via pipette. After the addition of enzyme, absorbance at 400 nm was measured for 5 min. All measurements were taken in triplicate. The final concentrations for assay components were 100 mM TAPS buffer (pH 8.5), 25.3 μM substrate, 504 μM NADPH, 5.04 mM MgCl_2_, 0.4 μM enzyme, and varying concentrations of inhibitor. Initial velocities were determined by linear regression over the linear range of the reaction using the Agilent Cary WinUV Software, and IC_50_ values were determined by plotting the log of anhydrotetracycline concentration against initial velocities v_0_ in GraphPad Prism 6.

### Statistics and reproducibility

Biochemical assays were performed in triplicate at a minimum. Statistical analysis for in vitro enzyme kinetics and inhibition assays were done using GraphPad Prism 6. All error bars shown depict standard error for three independent trials.

### Reporting summary

Further information on research design is available in the Nature Research Reporting Summary linked to this article.

## Supplementary information

Supplementary Information

Description of Additional Supplementary Files

Supplementary Data 1

Supplementary Data 2

Reporting Summary

## Data Availability

*Psuedomonas aeruginosa* Pa-3 assembly and tetracycline resistance genes have been deposited to NCBI under BioProject ID PRJNA615643. Source data underlying main text figures can be found in Supplementary Data 2.
