# Supplementary material for: Tetracycline-inactivating enzymes from environmental, human commensal, and pathogenic bacteria cause broad-spectrum tetracycline resistance
Source: Commun Biol. 2020 May 15;3:241. doi: 10.1038/s42003-020-0966-5 (PMC7229144; doi:10.1038/s42003-020-0966-5)
Supplement: Supplementary file 1 — Supplementary Information [file 42003_2020_966_MOESM1_ESM.pdf]

**Supplementary Information**

**Title:** Tetracycline-inactivating enzymes from environmental, human commensal, and pathogenic bacteria cause broad-spectrum tetracycline resistance

**Contents:**

**Supplementary Table 1:** Primers used in this study.

**Supplementary Figure 1:** Tet(X7) inactivates latest-generation tetracyclines by monooxygenation.

**Supplementary Figure 2:** Product of Tet(X7) hydroxylation of tigecycline coelutes with product of Tet(X) hydroxylation of tigecycline.

**Supplementary Figure 3:** Structural comparison of Tet(X7) and Tet(X) structures.

**Supplementary Figure 4:** Sequence comparison of clinically implicated *tet(X)* variants.

## Supplementary Figures

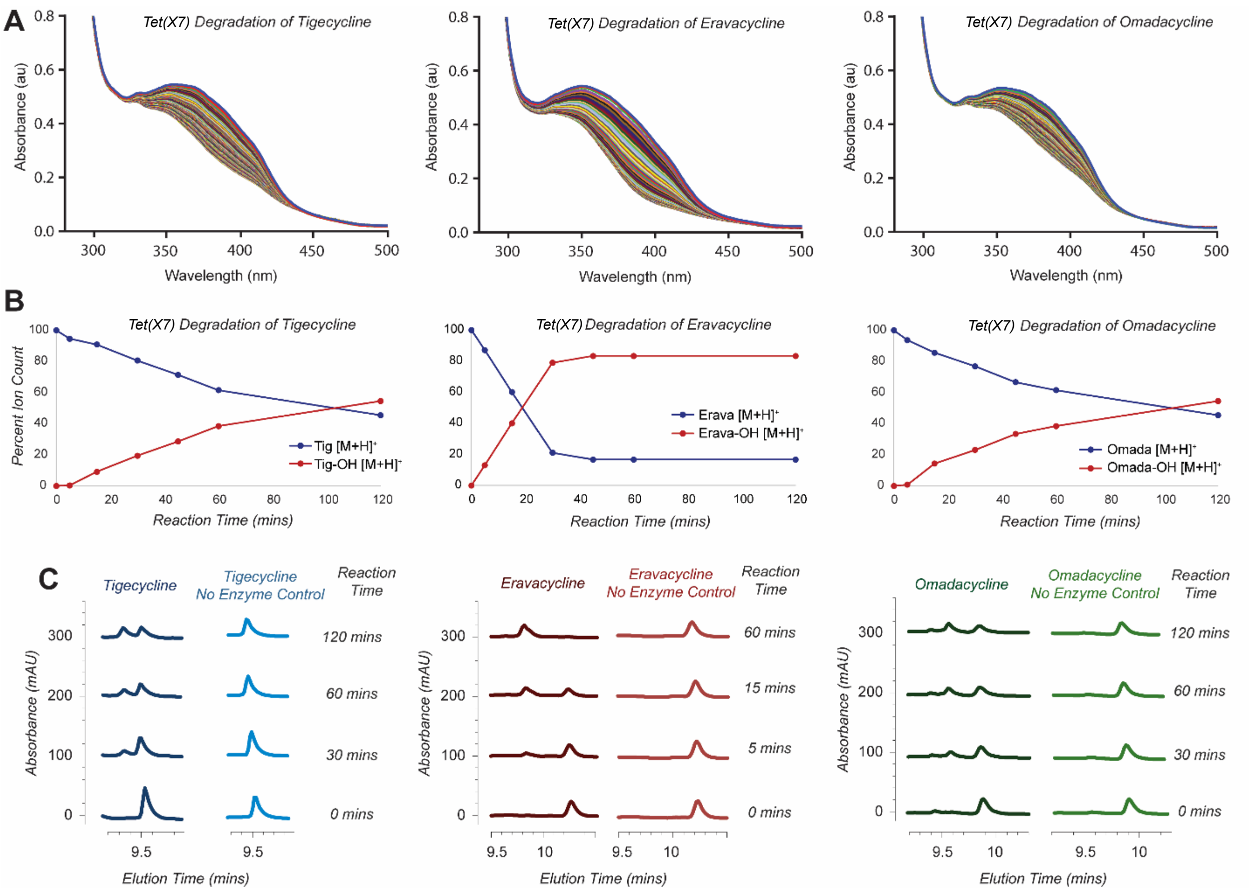


**Supplementary Figure 1: Tet(X7) inactivates latest-generation tetracyclines by monooxygenation.** (a) *In vitro* reactions containing purified Tet(X7), NADPH, and tigecycline, omadacycline, or eravacycline were assayed by absorbance scans taken at one minute intervals. The rainbow pattern depicts a spectral change over time. Decrease in absorbance over time from 360 nm to 400 nm indicates enzymatic disruption of the characteristic tetracycline β-diketone chromophore and consumption of NADPH. (b) In the case of tigecycline, omadacycline, and eravacycline, the product of enzymatic modification is consistent with monooxygenation. Plots show the relative extracted ion counts of the parent tetracycline (blue curves; *m/z* for $[M+H]^+$ equal to 586, 557, 559, respectively ) or the monooxygenated product (orange curves; *m/z* for $[M+H]^+$ equal to 602, 573, 575, respectively). (c) HPLC analysis with optical absorbance at 260 nm confirms enzyme-dependent degradation of tigecycline, eravacycline, and omadacycline by Tet(X7).

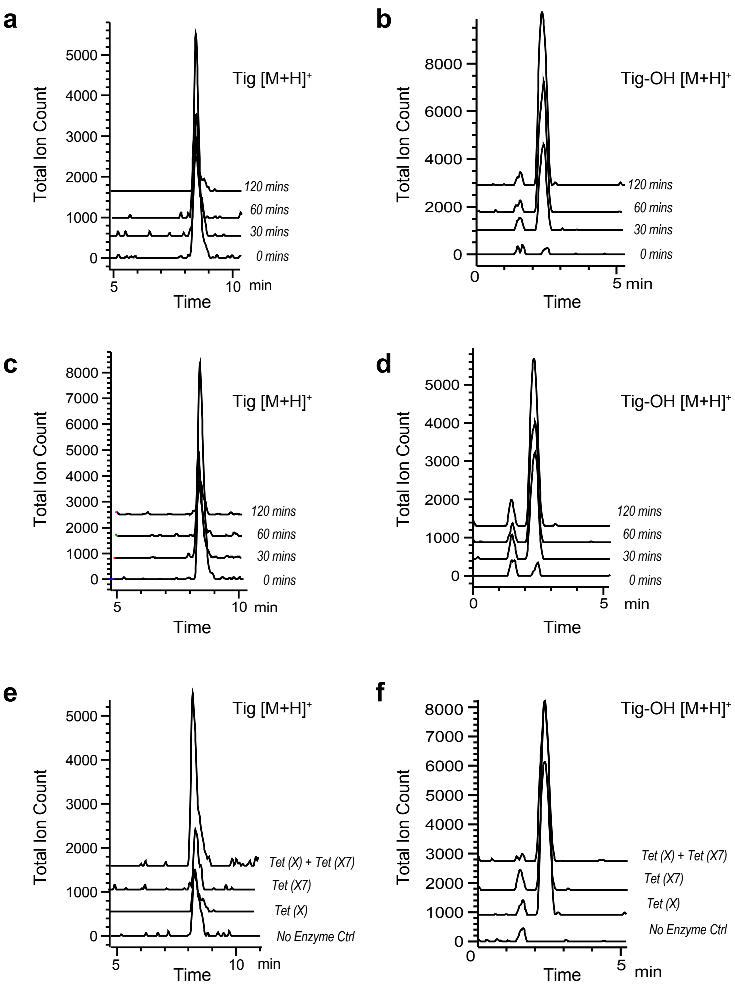


**Supplementary Figure 2: Product of Tet(X7) hydroxylation of tigecycline coelutes with product of Tet(X) hydroxylation of tigecycline.** *In vitro* reactions containing NADPH, tigecycline, and purified Tet(X) (a, b) or Tet(X7) (c, d) assayed by LC-MS indicate conversion of tigecycline (*m/z* for $[M+H]^+$ equal to 586) to monooxygenated tigecycline (*m/z* for $[M+H]^+$ equal to 602) over 120 minutes. Plots show extracted ion count LC-MS chromatograms for tigecycline (e) and monooxygenated tigecycline (f) from reactions with Tet(X) and Tet(X7) after 120 minutes. Signals were smoothed in ChemStation software using the “smooth all signals” function with a Gaussian fit of 0.05 min and a step size of 0.156 min. Also included are no enzyme controls and equimolar mixtures of the products of reactions with both enzymes. The products of Tet(X) and Tet(X7) monooxygenation of tigecycline coelute.

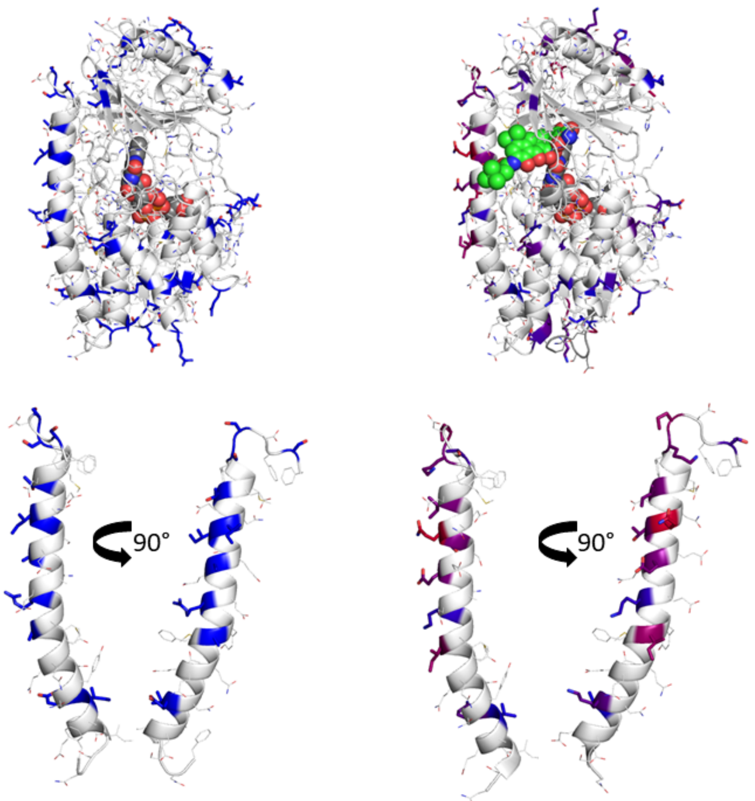


**Supplementary Figure 3: Structural comparison of Tet(X7) and Tet(X) structures.**

Top view: Two proteins are shown in cartoon (left: Tet(X7) and right: Tet(X)). Bound FAD is shown in grey (C-grey, O-red, N-blue) spheres in both the structures. The bound tigecycline in Tet(X) (PDB ID: 4A6N) is shown in green (C-green, O-red, N-blue) spheres. The conserved residues between two proteins are shown as lines and all different residues are shown in stick representation. In Tet(X7) (left), the divergent residues are colored as blue and in Tet(X), the divergent residues were colored based on the comparative physiological properties of residues in the two proteins. Blue represents the most similar substitution and the red represents the most contrasting substitution. Bottom view: The y-axis is rotated by 90° (counterclockwise) and the bridge-helix is shown in zoom.

**a**

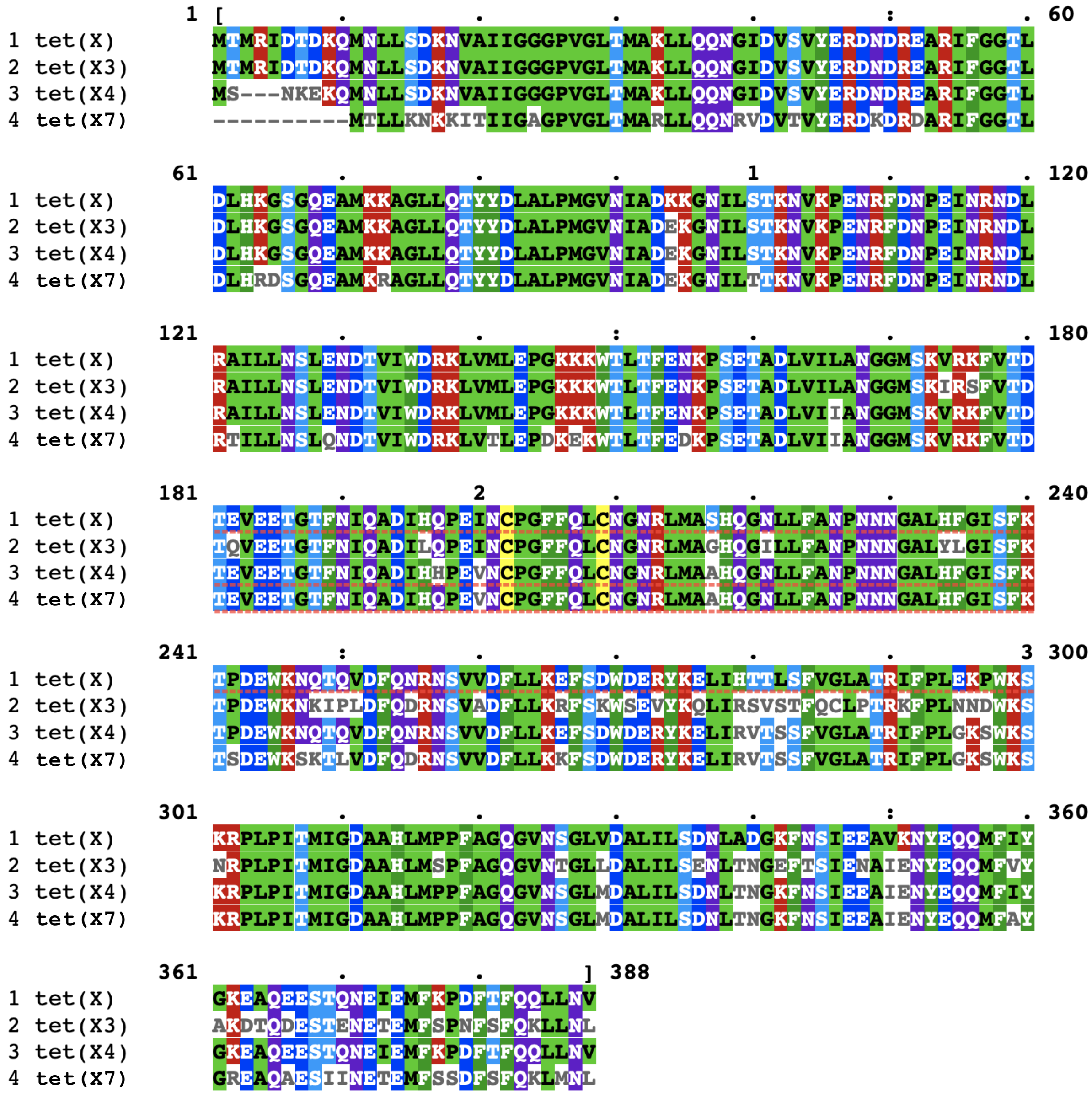


**b**

| | *tet*(X) | *tet*(X4) | *tet*(X7) | *tet*(X3) |
|---|---|---|---|---|
| *tet*(X) | 100 | 95.06 | 85.71 | 85.57 |
| *tet*(X4) | 95.06 | 100 | 88.89 | 85.45 |
| *tet*(X7) | 85.71 | 88.89 | 100 | 80.95 |
| *tet*(X3) | 85.57 | 85.45 | 80.95 | 100 |

**Supplementary Figure 4: Sequence comparison of clinically implicated *tet(X)* variants.** Multiple sequence alignment (A) and percent identity matrix (B) of clinically implicated *tet*(X) variants. Residues in multiple sequence alignment are colored when identical to *tet*(X) based on amino acid property according to default MView color palette[63].

**Supplementary Table 1 Primers used in this study**

| **Primer sequence (5' → 3')** | **Designed specificity** |
|---|---|
| ATGACTTTGCTAAAAAATAAAAAAATTA | TE_7F_Contig_3 (452_1588) |
| TTATAGATTCATTAGTTTTTGGAATGA | TE_7F_Contig_3 (452_1588) |
| ATGAATTTACTAAACAATAAAAAAGTTACA | TG_0402_Contig_27 (425_1561) |
| TTATAGATTCATTAGTTTTTGGAATGA | TG_0402_Contig_27 (425_1561) |
| ATGACAATGCGAATAGATACAGACA | TE_0402_Contig_1789 (2156_3322) |
| TTATACATTTAACAATTGCTGAAACG | TE_0402_Contig_1789 (2156_3322) |
| ATGACTTTGCTAAAAAATAAAAAAATT | TE_0402_Contig_1211 (2189_3325) |
| TTATAGATTCATTAGTTTTTGGAATGA | TE_0402_Contig_1211 (2189_3325) |
| ATGACAATGCGAATAGATACAGACA | TG_0402_Contig_45 (861_2027) |
| TTATACATTTAACAATTGCTGAAACG | TG_0402_Contig_45 (861_2027) |
| ATGACAATGCGAATAGATACAGACA | TG_0401_3a_Contig_236 (181_1347) |
| TTATACATTTAACAATTGCTGAAACG | TG_0401_3a_Contig_236 (181_1347) |
| ATGACTTTACTAAAACATAAAAAAATTACA | TE_6F_Contig_7 (1101_2237) |
| TTATAGATTCATTAGTTTTTGGAATGA | TE_6F_Contig_7 (1101_2237) |
| ATGACTAGCGATAAGAACCCT | S08_TE.2:1046-1630 Contig:2 |
| TCATTCGTATTCTGGTAGCG | S08_TE.2:1046-1630 Contig:2 |
