# Supplementary material for: Tetracycline-inactivating enzymes from environmental, human commensal, and pathogenic bacteria cause broad-spectrum tetracycline resistance
Source: Commun Biol. 2020 May 15;3:241. doi: 10.1038/s42003-020-0966-5 (PMC7229144; doi:10.1038/s42003-020-0966-5)
Supplement: Supplementary file 2 — Description of Additional Supplementary Files [file 42003_2020_966_MOESM2_ESM.pdf]

Description of Additional Supplementary Files

File Name: **Supplementary Data 1**
Description: Accession numbers for resistance enzymes described in this work.

File Name: **Supplementary Data 2**
Description: Source data underlying main text figures and graphs.
